# The Effect of Anatomical Reduction on Functional Outcomes in Femoral Neck Fracture: A Novel Modified Garden Index

**DOI:** 10.7759/cureus.19863

**Published:** 2021-11-24

**Authors:** Anil Agar, Ali Utkan

**Affiliations:** 1 Orthopedic and Traumatology, Saglik Bilimleri University, Kanuni Sultan Suleyman Training and Research Hospital, Istanbul, TUR; 2 Orthopedics and Traumatology, Ankara City Hospital, Ankara, TUR

**Keywords:** harris hip score, garden alignment index, anatomical reduction, cannulated screw, femoral neck fracture

## Abstract

Background: Femoral neck fracture is a common cause of morbidity in the younger population with potentially poor prognosis and functional results. The purpose of this study was to evaluate how the success of anatomic reduction affects hip functional outcomes by using a new numerical index modified from the Garden index.

Methods: Twenty-six patients who underwent closed reduction and internal fixation by means of three cannulated screws due to femoral neck fracture between 2010 and 2014 were scanned retrospectively and included in the study. Harris hip scores of the patients at nine to 12 months were evaluated using the new index modified from Garden which is the sum of the differences from the ideal Garden index calculated on early postoperative anteroposterior and lateral pelvis radiographs.

Results: The mean hip score was 73.1 (± 16.4), the minimum score was 32, the maximum score was 93. Postoperative anteroposterior radiographs revealed a mean Garden alignment index of 153.6°, the lowest value was 135° and the highest value was 168°. In the lateral radiographs, the mean Garden alignment index was 173.1°, the lowest value was 160° and the highest value was 178°. The mean value of the novel index was found as 14.2°, the lowest value was 2°, and the highest value was 40°. There was an inverse correlation between the novel index we defined and the Harris hip score (p<0.01).

Conclusion: In femoral neck fractures, the success of anatomic reduction is an important factor affecting the patient’s functional outcome. We believe the new reduction index we proposed will contribute to literature comparing the success of reduction in femoral neck fractures.

## Introduction

Femoral neck fracture is a common cause of morbidity and mortality in the elderly and one of the challenging fractures for an orthopedic surgeon in the younger population with a potentially poor prognosis [1,2]. This treatment aimed to restore the patients’ pre-fracture functional status as soon as possible without leading to any complications [3]. Although internal fixation of the fractured femur is associated with various complications, the current practice supports early fixation with anatomic reduction, and arthroplasty is mainly reserved for older patients [4].

Functional outcomes of femoral neck fractures depend on individual patient factors such as ambulatory condition, age, mental functions, other systemic diseases, and fracture-related factors such as fracture type, degree of displacement, and duration up to the operation. Femoral neck fractures cause considerable damage to the ascending cervical arteries of the extracapsular arterial ring. The surgeon has limited control on initial vascular damage supplying the femoral head [5,6]. It is clear that the treatment of femoral neck fractures is surgical, but there is disagreement on which surgical procedure to perform. As restoration of the blood supply would be prohibited by inappropriate reduction, both anatomic reduction and the maintenance of the reduction are important. Treatment of the fracture should begin at the earliest time. Treatment preference depends on the type of fracture and the patient’s age. Several implants such as dynamic condylar screws, compression screws, sliding hip screws, and locked plates are available. Stable fixation reduces the possibility of osteonecrosis and nonunion [7-9].

Garden developed an alignment index to define the amount of reduction based on the alignment of the trabecular structures on anteroposterior and lateral x-ray images of the hip [10]. However, his index only classifies the position of reduction into one of four groups so it is a categorical index. The purpose of our study was to investigate how anatomic reduction affects functional results by using a new numeric index that can assist in evaluating the accuracy of reduction.

## Materials and methods

The patients of 20 to 65 years who were treated by closed reduction and internal fixation with three cannulated screws for their femoral neck fractures between April 2010 and January 2014 were scanned retrospectively and included in the study. The institutional review board of the hospital approved the study (number: 803/2014). The patients younger than 20 years, older than 65 years, patients who had undergone hip surgery previously, patients with pathologic fractures, and patients whose fractures could not be reduced by closed methods were excluded from the study. 

After the rapid assessment and treatment of the patients in the emergency room, a detailed orthopedic physical examination was performed and necessary radiographs were obtained. In patients who presented with high-energy trauma, priority was given to concomitant life-threatening pathologies. The etiologies of the fractures were noted. The fractures were classified according to the Garden classification [11]. Patients underwent surgery as soon as possible after the completion of pre-operative assessment and time to operation was recorded.

In supine position, after prepping and draping the patient, the fracture was reduced by the Leadbetter technique [12]. Manual traction was maintained throughout the operation by an assistant. Anteroposterior and lateral views of the image intensifier were assessed. If the reduction was accepted as satisfactory, three parallel K wires were inserted through the femoral neck using an aiming device. Either triangular or inverted triangle configuration was used according to the surgeon’s preference. After drilling over the wires, proper length 6.6 mm cancellous screws were inserted percutaneously. Washers were used almost in each screw.

Thromboembolism prophylaxis with low molecular weight heparin was started postoperatively and continued for 30 days. As antibiotic prophylaxis, cefazolin sodium was administered 30 minutes before surgery and continued for the first 24 hours postoperatively. In the early postoperative period, all patients except those who could not be mobilized due to additional orthopedic or systemic injuries began quadriceps exercise and active straight leg lifting exercise on the first postoperative day and were encouraged to walk as soon as possible without weight-bearing to operated leg The patients were allowed to partially bear weight after 45 days. Full weight-bearing was allowed individually according to clinical and radiographic findings.

Patients were followed at appropriate intervals and clinical and radiographic findings were noted. The Harris hip scores of patients were obtained at 12 months. However, in two patients whose fractures were not healed, the hip scores obtained at their last clinical follow-up which was at nine months were taken into consideration.

**Figure 1:**
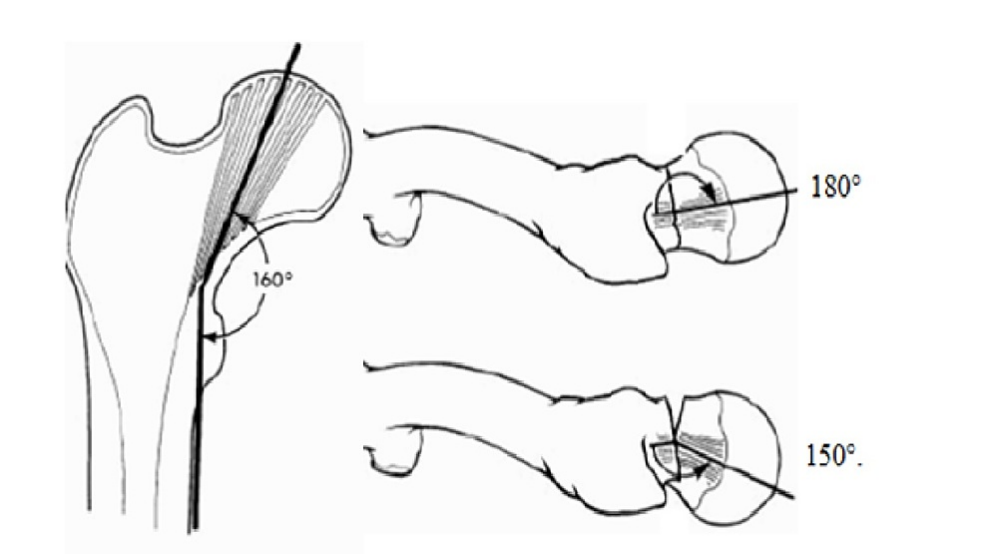
Garden alignment index on the anteroposterior and lateral radiographs

**Figure 2:**
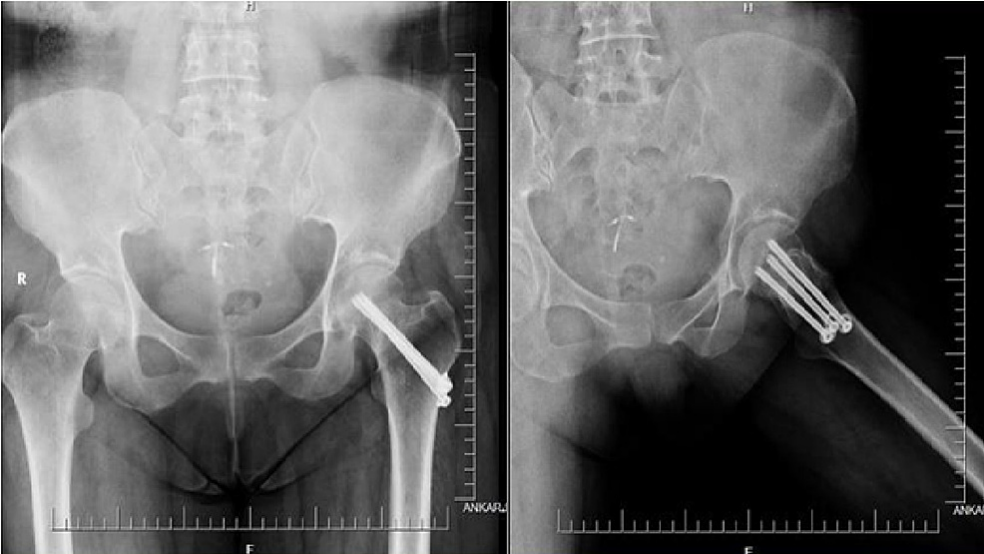
Postoperative anteroposterior and lateral radiographs of a 43-year-old female patient

Immediate postoperative anteroposterior and lateral radiographs of the patient were evaluated according to Garden’s alignment Index (Figures 1, 2) [10]. The index is used to evaluate the degree of reduction of the femoral neck fractures on anteroposterior and lateral planes. Normally, the angle between the primary compressive trabeculae of the neck and diaphysis is 160° on an anteroposterior radiograph, and this angle is usually called as Garden index. The major trabeculae on the femoral neck axis extend at an angle of 180° on true lateral radiograph. These angles change due to the fracture and restoration of them to ideal values are aimed by reduction.

In Garden’s original study, the alignment indexes were evaluated in four categories. The reduction was considered good if the slip was less than 2 mm, and the change in angles was 0-5° on the anteroposterior radiograph and less than 10° on the lateral radiograph. On the anteroposterior radiograph, if the displacement was 2-5 mm, there was 5° varus or 5-20° valgus, and 10-20° angulation on the lateral radiograph, the reduction was accepted as moderate. The reduction was considered worse if there was a displacement of more than 5 mm between the fracture ends more than 5° varus or more than 25° valgus on the anteroposterior radiograph and more than 20° lateral angulation. Unacceptable varus reduction below 25° and excess valgus exceeding 180° increased the risk of avascular necrosis and degenerative changes due to mismatch between the hip joint surfaces. If the femoral head was in an antevert or retroverted position in lateral and the angle was below 150° in lateral x-ray, there was an unstable and improper reduction [10].

In our study, early postoperative radiographs were used to evaluate the success of reduction. We first calculated the difference between the alignment angle after reduction and 160° on anteroposterior radiographs. Then we calculated the difference between the alignment angle after reduction and 180° on lateral radiographs. The sum of these two values provided us with the novel index value.

The functional status of the patients was evaluated and scored according to Harris’ Hip Function Assessment. The hip score is a scale developed by Harris in 1969 after applying arthroplasty after hip dislocation and acetabular fracture. The Harris hip scale consists of four subheadings: pain, function, range of motion, and absence of deformity. The maximum score is 100 [13]. The patient’s functional scores were obtained at a one-year follow-up except for two patients that underwent total hip arthroplasty. In these patients, Harris hip scores obtained at the ninth month were recorded.

Statistics

The IBM Statistical Package for the Social Sciences (SPSS) 22.0 program (Istanbul, Turkey: IBM Corp.) was used for statistical analysis while evaluating the findings obtained in the study using a t-test, one-way analysis of variance (ANOVA), and chi-square test. While evaluating the study data, the Mann-Whitney U test compared parameters that did not show a normal distribution of descriptive statistical methods (mean, standard deviation) between two groups. In addition, the relationships between continuous variables were investigated using Pearson and Spearman's rank correlation analysis to examine the relationships between parameters. Significance was evaluated at the p <0.05 level. A linear regression analysis was conducted to determine the effect of the modified Garden index on the Harris score.

## Results

The mean age of the patients was 47.8 (± 9.9) years, the youngest age was 26 years, and the oldest age was 64 years. Twelve (46%) patients were female and 14 (54%) were male (Tables 1, 2). Twenty-three (88.4%) of the patients experienced a simple fall and three (11.6%) had a femoral neck fracture resulting from high-energy trauma. The average duration from hospitalization to operation was 15 hours, ranging from a minimum of five hours to a maximum of 51 hours. The minimum follow-up period was nine months, and the longest follow-up period was 52 months (mean 21.6 months). Additional orthopedic injuries were present in six patients (23%). These included lumbar vertebra transverse process fracture, sacrum fracture, scaphoid fracture, distal radius fracture, humerus proximal fracture, and ipsilateral medial malleolar fracture. According to the Garden fracture classification, one patient (3.8%) had type 1, eight (30.7%) had type 2, 11 (42.3%) had type 3, and six (23%) had type 4 fractures (Tables 1, 2).

**Table 1:** The patients' demographics and functional outcomes

Factors			Number (n)	Percentage (%)
Gender		Male	14	54
		Female	12	46
Garden classification		Type 1	1	3.8
		Type 2	8	30.7
		Type 3	11	42.3
		Type 4	6	23
Harris score	Excellent		7	26.9
	Very good		11	42.3
	Good		1	3.8
	Moderate		5	19.2
	Poor		2	7.7

**Table 2:** The patients' demographics and mean (±SD)

Factors	Mean (±SD)
Age (year)	47.8 (±9.9)
Duration from hospitalization to operation (hour)	15.3 (±10.6)
Garden index - anteroposterior	153.6 (±7.5)
Garden index - lateral	173.1 (±5.1)

At postoperative anteroposterior radiographs mean Garden alignment index was 153.6° (the lowest value was 135° and the highest value was 168°). It is spotted that in three cases it was outside the 140-160° range. In the lateral radiographs, the mean Garden alignment index was 173.1° (the lowest value was 160° and the highest value was 178°). The differences in the Garden alignment index from 180° on lateral radiographs were calculated. The average difference was 6.8°, the lowest was 2° and the highest was 20°. The new index was calculated for each patient by adding the differences. The mean value was 14.2°, the lowest value was 2°0 and the highest value was 40°.

In one of the patients, a pedicled sartorius graft was applied because of non-union in the ninth month. This patient underwent total hip arthroplasty in another hospital. Another patient underwent total hip arthroplasty in the tenth month. In another case, one of the screws was removed at postoperative tenth month because of screw migration due to impaction, but fortunately, healing was good and no additional surgical intervention was performed thereafter. Despite radiological findings of avascular necrosis were noticed later in four patients, no additional surgical intervention was performed until their last follow-up.

The mean Harris hip score at nine to 12 months was 73.1 (± 16.4), the minimum score was 32 and the maximum score was 93. The Harris hip score was considered excellent in seven patients, very good in 11 patients, good in one patient, and moderate in five patients. The results were poor in two patients that underwent total hip arthroplasty later on (Tables 1, 2). There was a significant inverse correlation between Harris hips scores and the novel index. Furthermore, type of fracture and duration up to operation were inversely correlated with Harris hip scores (Table 3).

**Table 3:** Correlation analysis of duration up to the operation, the novel index, and Garden classification of fractures with Harris hip score *p<0.01

	Harris hip score	
	r-value	p-value
Duration from hospitalization to operation	-0.694	0.001*
Novel index	-0.541	0.004*
Garden classification	-0.605	0.001*

## Discussion

Despite progress in medicine, the treatment of femoral neck fractures in young patients remains a challenge for orthopedic surgeons. Complications that adversely affect the hip functions of the patients are observed not infrequently. Anatomic restoration of the joint as soon as possible after the trauma prevents the impending complications. Because of increased traffic and work-related accidents, such events are more frequent in the young age groups and men [14]. In the present study, 54% of the patients were male consistent with the literature, but contrary to the literature, 88.4% of the patients had fracture after a simple fall.

The Garden classification is the most widely accepted and commonly used classification for femoral neck fractures. The classification is simple and effective in predicting prognosis compared to other classifications. Majernicek found that 53 of 64 cases were Garden type III-IV, and 11 were type I-II [15]. In Parker’s report on 242 cases, 4.6% were type I, 21.9% were type II, 23.5% were type III, and 50% were type IV [16]. Lee et al. reported 116 patients, 104 (90%) cases were Garden types I and II, and 12 (10%) were Garden types III-IV [17]. In the current study, according to the Garden fracture classification, one patient (3.8%) had type 1, eight (30.7%) had type 2, 11 (42.3%) had type 3, and six (23%) had type 4 fractures. This result was consistent with the literature.

Avascular necrosis is one of the devastating complications that might occur after a femoral neck fracture and is reported that 25% in those who underwent surgery in the first 12 hours, 30% in those performed in 13-24 hours, 40% in those conducted after a delay of 25-46 hours, and 100% in those performed a week later [18]. Swiontkowski reported that the risk of osteonecrosis and non-union is lower in those who underwent surgical reduction and rigid internal fixation during the first 12 hours [8]. In this study, the average duration up to the surgery was 15 hours and ranged from the lowest five hours to the highest 51 hours. In the present study, the Harris score deteriorated when the duration up to the surgery increased; this correlation was statistically significant (p<0.01) (Table 3). However, the rate of avascular necrosis was found to be consistent with the literature (6/26; 23%) [18,8].

After surgical treatment of the femoral neck fracture, the goal is to obtain a painless and functional hip joint. Seçinti et al. reported 35 of the 46 (76.1%) patients were excellent and good, 11 (23.9%) were fair and poor [19]. In Tükenmez et al., based on the results of the hip score, 16 (16%) cases were very good, 17 (41.5%) cases were good, five (12.2%) cases were moderate, and three (7.3%) patients had poor results [20]. Karsan et al. recorded hip scores in 26 patients (58.8%) as good, 13 (29.7%) medium, and five (11.5%) as bad [21]. Kayali et al. found that 69% of 32 patients had good and 31% had bad results according to Harris score [22]. Ibraheem and Massoud observed limited movement in 26 (96.3%) patients, with full movement in 27 (96.3%) patients [23]. In the present study, the mean Harris score for the hip was 73.1 (± 16.4), the minimum was 32 and the maximum score was 93. Seven patients scored excellent, 11 patients scored very good, one patient scored good, five patients scored moderate, and two patients scored poor.

Anatomic reduction is very important in femoral neck fractures. Garden developed an index to define acceptable reduction based on the arrangement of trabecular structures on the anteroposterior and lateral images. According to Garden, the angle between the primary compressive trabeculae of the neck and the diaphysis is 160° on anteroposterior radiographs. On lateral radiograph, major trabeculae of the femoral neck extend at an angle of 180°. A Garden alignment index between 150-160° on anteroposterior radiograph and 150-180° on lateral radiograph reduces the risk of avascular necrosis and forthcoming joint degeneration. If after reduction these angles are measured outside these ranges, the reduction should be accepted as unstable and inappropriate, either manipulation should be repeated or the management should be changed.

Studies have shown that varus deformity due to the collapse of the femoral neck is a critical problem [24,25]. Femoral neck shortening caused by collapse affects the functional results of patients adversely. In patients with impaired alignment in the Garden index, late lateral collapse is the most common cause of these results [24]. Kyle determined that satisfactory long-term effects can be achieved by providing anatomical reduction and fixation in patients treated with multiple screw fixation methods [25]. Medda et al. stated that there was no difference between open reductions and closed reductions, indicating that the quality of the reduction was more important than timing alone and that all conditions for optimal reduction and fixation should be appropriate [26]. On our patients’ postoperative radiographs, the Garden alignment indexes were measured on the anteroposterior radiographs, and the differences from 160° were calculated. In the lateral radiographs, the Garden alignment indexes were measured and the differences from 180° were calculated. Then these two values were summed up to obtain the novel index. In the present study, there was an inverted correlation between Harris scores of the patients and their novel scores (p<0.01).

There are several limitations of this study. The study was a retrospective study conducted at a single institution on a small number of patients who had only relatively small term follow-up. The effect of this index on long-term functional outcomes in femoral neck fractures is not known.

## Conclusions

Although the treatment of femoral neck fractures is a challenge to orthopedists, it is possible to obtain satisfactory results by closed reduction and fixation with cannulated screws. The type of fracture according to Garden, duration up to the operation, and the success of reduction are the critical factors affecting the patient’s outcome. We believe that the novel index we have proposed could assist in evaluating the accuracy of reduction and would contribute to the literature.
